# Identifying spatial clustering of diarrhoea among children under 5 years across 707 districts in India: a cross sectional study

**DOI:** 10.1186/s12887-023-04073-3

**Published:** 2023-05-30

**Authors:** Koustav Ghosh, Atreyee Sinha Chakraborty, Shoummo SenGupta

**Affiliations:** 1 Gokhale Institute of Politics and Economics, Pune, India; 2 Population Research Centre Baroda, Gujarat, India; 3 International Institute for Population Sciences (IIPS), Mumbai, India

**Keywords:** Mortality; Morbidity, Under-five children, Spatial, Autocorrelation, Sustainable Development Goal

## Abstract

**Background:**

Diarrhoea is one of the leading reasons for under-five child mortality and morbidity across the globe and especially in low- and middle-income countries like India. The present study aims to investigate and identify the spatial clustering and the factors associated with diarrhoea across 707 districts of different states in India.

**Methods:**

This study used National Family Health Survey-4 & 5 (2015–16 & 2019–21) data in India. Spatial analysis software i.e., ArcGIS and GeoDa including Moran’s statistics have been applied to detect the spatial prevalence and auto-correlation of diarrhoea among neighbourhood districts. Bivariate analysis with a chi-square test and logistic regression has been performed to identify the factors associated with the morbidity condition.

**Results:**

The result shows out of 2,23,785 children, 7.3 percent children suffer from diarrhoea in India. The prevalence is highest in Bihar (13.7%) and lowest in Lakshadweep (2.3%). Around 33 percent of districts have reported more than the national average level of diarrhoea prevalence. The study also found a medium to high level of autocorrelation with 0.41 Moran’s Index value and detected 69 hot-spots districts mostly from Maharashtra, Bihar, Odisha, and Gujarat. The study has also found, with an increase in children’s age as well as mother's age the prevalence of the disease decreases. The prevalence is more among male children than females. Underweight [OR = 1.08, 95% CI (1.03–1.13)] children have a greater risk of suffering from diarrhoeal diseases. The odds of children living in a pucca house [OR = 0.89, 95% CI (0.68–1.16)] are less likely to suffer from diarrhoea. On the other hand, rich economic status [OR = 0.91, 95% CI (0.86–0.97)], reduce the risk of such morbid conditions.

**Conclusion:**

The study recommends targeting the hot-spot districts with high prevalence areas, and district-level interventions by improving housing type and child nutrition status, which can help to prevent diarrhoeal diseases among children in India. Thus, the identification of hotspot districts and suggested policy interventions by the current study can help to prevent childhood mortality and morbidity, as well as to achieve the target given by Sustainable development Goals 3.2.

## Background

Global Burden of Diseases (GDB, 2017) reported that diarrhoea is one of the leading causes for under-five mortality and morbidity across the globe [1]. The latest report of The World Health Organisation (2017) states that among death in children under 5 years of age, 8% could be attributed to diarrhoea [2]. In the year 2017, globally 1.7 billion children suffered from diarrhoea, and out of that, more than half a million died [3]. It accounts for the second leading cause of under-five mortalities in the world [3]. Diarrhoeal diseases eventually lead to other serious morbidities among children. Precisely, diarrhoea is more prevalent among children in second year of their life [4, 5]. The United Nations International Children’s Emergency Fund (UNICEF) revealed that among death in children under 5 years of age, 45% could be associated to malnourishment. Pneumonia (15%), diarrhoea (8%) and malaria (5%) account for three major causes of death among under 5 children.[4]. However, in developing countries, diarrhoeal disease is the major cause of malnutrition, contributing to the third major cause of under 5 mortalities [3, 4]. Diarrhoea has been associated with frequent hospital visits and admissions. In the developing countries, on average three times a year, children suffer from diarrhoea [6–8].

India, Nigeria, Congo, Pakistan, and China together contributed to half of the diarrhoeal deaths of children (4.249 million) in 2008 [7]. In 2005, diarrhoea alone contributed to 14% of the total deaths among children under-five years of age in India [9, 10]. The National Family Health Survey shows that the prevalence of childhood diarrhoea had increased from 9 percent to 9.2 percent from 2006 to 2016 [11, 12]. Interestingly, the prevalence has decreased to 7.3 percent in 2021 [13]. But still, it is the third most common disease responsible for under-five mortalities in the country [14].

There are some studies in contemporary literature that identify the spatial clustering and prevalence of diarrhoea in India. In a study based on the National Family Health Survey-4 (NFHS-4, 2015–16) 71 hot-spot districts have been identified with a Moran’s Index of 0.38 [15]. Furthermore, some other studies have shown the mother’s age, the age of the child, social class, religion, residence, and wealth index have an influence on childhood diarrhoeal cases in India [15–17].

The present study aims to investigate the spatial prevalence and factors associated with diarrhoea in districts of India among children, along with hot-spot analysis. We have used spatial analysis software to show the spatial prevalence and examined the clustering of diarrhoea by incorporating multiple linked risk factors which have not been reported. The analysis is based on recently published NFHS-5 (2019–21) data.

These results will give detailed knowledge about spatial prevalence, and it will help in the identification of the hot-spot districts with higher prevalence of diarrhoeal cases among children in India.

This information will help at the district level in policy formulation and advocacy, which can play an important role in decreasing child morbidity and mortality. This assessment is also very important for India given the various global health efforts e.g., the United Nation’s Sustainable Development Goals (SDG-3.2: ending deaths of new-borns and under-five children by 2030) [18] and Global Action Plan for Pneumonia and Diarrhoea (GAPPD: ending preventable pneumonia and diarrhoea deaths by 2025) [19]. The country has already set its target to achieve SDGs goals of decreasing under-five child mortality to 25/1000 live births in 2030 (SDGs) which is 42/1000 live births as per the National Family Health Survey (2019–21) [13, 18].

## Methods

### Data source

The present study uses National Family Health Survey (NFHS-5) data of India, 2019–21. This survey covered 36 states/UTs including 707 districts of India [13]. It also uses NFHS-4 (2015–16) data as well which has covered 36 states/UTs including 640 districts. This survey is used to be conducted under the supervision of the Ministry of Health & Family Welfare (MoHFW), Government of India. The Institute for Population Sciences (IIPS), Mumbai, was the nodal agency for NFHS-4&5. The study is based on multistage stratified sampling. It provides an estimation of anthropometric, clinical and biochemical (CAB) components along with the prevalence of malnutrition, hypertension, diabetes, and HIV through the biometric measurement.

In the NFHS survey, the information on diarrhoea among children was collected from mothers of under 5 child/children from the sampled household. The question was asked whether her child/ children had suffered from diarrhoeal diseases before the last two weeks of the survey. The response was recorded as ‘0′ no’ and ‘1′ yes. However, to mention here, the accuracy of these replies depends on the accuracy of the mother’s recall. Two weeks of recall was thought to be small enough to minimize the recall bias. In addition, the illnesses reported are based on the mother's perception and without spot validation by a medical person.

The study was restricted to living children of less than 60 months who lived with their mothers. The missing cases were also taken out of the dependent variable. Finally, a total of 2,23,785 children were selected for the final analysis. The dataset is freely available for download from https://dhsprogram.com/data/.

### Statistical analysis

The present study uses the spatial analysis software ArcGIS 10.8 and GeoDa 1.18 to understand the geographical variation and spatial clustering of diarrhoea across the 707 districts in India. Exploratory Spatial Data Analysis (ESDA) has been attempted to identify the existence of clustering, spatial auto-correlation, and spatial heterogeneity in the prevalence of childhood diarrhoea [20]. Geographical Information System (GIS) and spatial statistical techniques based on ESDA are useful in describing and representing spatial distribution, detecting hot-spot and cold-spot areas, and suggesting spatial regimes or other forms of spatial heterogeneity [20, 21]. Additionally, we have used the multilevel logistic regression model to know the most responsible factors for diarrhoea and contextual correlates with the help of STATA-14. We had prepared a logistic model (not shown), which makes the Hosmer–Lemeshow chi-square insignificant (*p* = 0.6) which means that the model is appropriate for logistic regression. Further, after taking the districts construction of a multilevel logistic model gives a significant intra-cluster correlation coefficient (ICC), which suggests that a multilevel model is appropriate and best fitted for our study [22]. In that model, we used various explanatory variables as per the previous study (i.e., environmental, socio-economic, and children related demographic) [15, 23].

### Spatial analysis

In order to analyse hot-spot in spatial tools the scale measure is important as it specifies the distance value of the map [21, 24]. For that, the spatial weight matrix (W) is essential for the computation of the spatial autocorrelation among districts of the study area. To construct the matrix, we need to define neighbourhood by assigning spatially contiguous weights (queen’s weight) that include all common points [21]. The spatial autocorrelation among the districts, can be shown by using Moran’s I and Local Indicators of Spatial Autocorrelation (LISA) is used to measure the extent of autocorrelation among the neighbourhood districts. Moran’s Index helps to understand the intensity of clustering (Z-score) by increasing the distance. The output of the z-score helps to determine the intensity of clustering. In the present study, LISA statistics were used to detect the hot-spot area across the districts considering the percentage of children suffering from diarrhoea [15] in the study area. The LISA values consent to the computation of its similarity with its neighbour districts and also test the level of significance (*p* values) for each location. Moreover, the LISA map portrays the results of five scenarios- a cluster with high values (high-high or hot spot), a cluster with low values (low-low or cold spot), outlier in which a high value which is surrounded by a low value (high-low), an outliers in which a low value is surrounded by a high value (low–high) and no significant local autocorrelation.

## Results

### Geographical variance of the prevalence of diarrhoea among 0–5 aged children in India

Table 1 represents the geographical variation of diarrhoea among children under-fives years at the state/UTs level in India (2019–21). At the national level, 7.3 percent of children under-five years of age suffer from diarrhoea. It is higher among children living in rural (7.7%) residences as compared to urban (6.2%) residents. Prevalence of diarrhoea is high in Bihar (13.7%) followed by Delhi (10.6%), Meghalaya (10.5%), Odisha (9.7%), and Maharashtra (8.9%). On the contrary, Lakshadweep (2.3%), Dadra & Nagar Haveli (2.7%), and Goa (3.2%) have reported lower prevalence. In the rural residence the diarrhoea prevalence has been reported from the range lowest in Chandigarh (0%) to highest in Bihar (13.9%). For females, the range is from 0.96 percent (Lakshadweep) to 13.74 percent (Ladakh).

**Table 1:** Prevalence of diarrhoea among 0–5 aged children in India and states/UTs, 2019–21

**State/UT’s**	**Urban**	**Rural**	**Total**	**Total Sample (N)**
Bihar	12.65	13.91	13.74	19,912
Delhi	10.57	11.99	10.62	2,852
Meghalaya	11.9	10.25	10.47	6,368
Odisha	10.28	9.58	9.68	8,153
Maharashtra	6.57	10.69	8.92	9,252
Ladakh	13.74	7.16	8.52	515
Gujarat	5.67	9.76	8.22	9,532
Telangana	5.46	8.62	7.38	7,100
Andhra Pradesh	6.24	7.61	7.22	2,740
Jharkhand	6.53	7.34	7.21	9,634
West Bengal	5.87	6.69	6.47	5,479
Madhya Pradesh	7.22	6.23	6.45	15,516
Tripura	3.93	6.88	6.21	1,986
Rajasthan	5.39	6.28	6.1	14,138
Andaman & Nicobar Island	6.36	5.17	5.72	441
Jammu & Kashmir	3.35	6.29	5.61	5,750
Manipur	5.52	5.63	5.59	3,128
Uttar Pradesh	5.17	5.68	5.57	33,768
Sikkim	9.32	3.35	5.56	605
Assam	2.74	5.8	5.46	10,276
Karnataka	4.61	5.64	5.25	8,140
Arunachal Pradesh	4.27	5.27	5.13	5,414
Haryana	4.85	5	4.96	6,634
Punjab	6.03	4.3	4.92	5,400
Himachal Pradesh	3.55	4.87	4.71	2,560
Uttarakhand	5.12	4.08	4.41	3,644
Chandigarh	4.4	0	4.36	169
Mizoram	4.84	3.74	4.3	2,400
Kerala	4.22	4.32	4.28	2,707
Tamil Nadu	3.58	3.82	3.71	6,370
Puducherry	2.89	5.67	3.69	757
Chhattisgarh	3.3	3.72	3.64	8,098
Nagaland	1.53	4.13	3.44	2,933
Goa	2.1	5.01	3.22	366
Dadra & Nagar Haveli	2.32	2.95	2.65	772
Lakshadweep	0.96	6.16	2.27	276
**India**	**6.17**	**7.73**	**7.31**	2,23,785

**Fig. 1:**
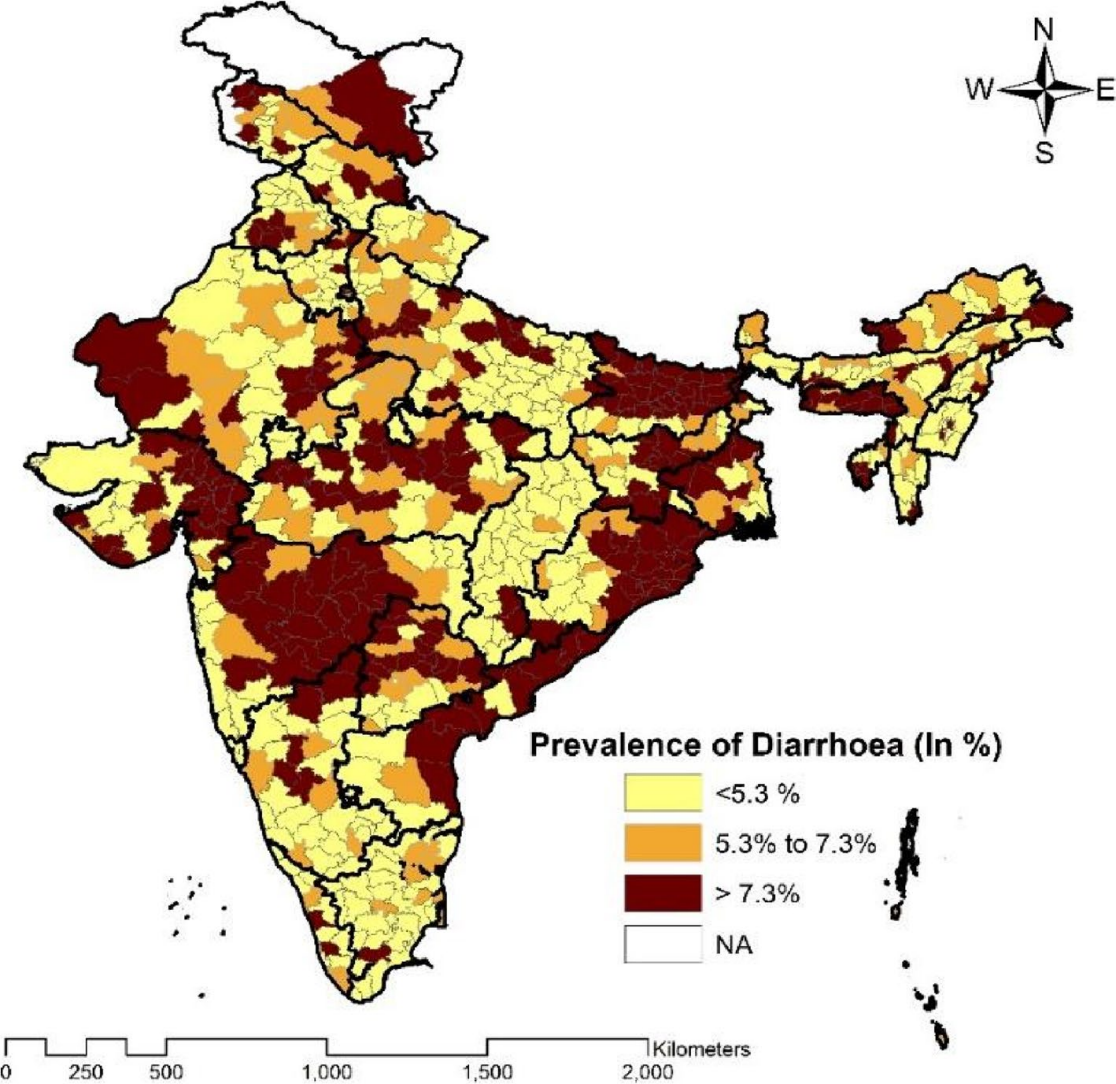
Spatial prevalence of diarrhoea among children aged 0–5 years in districts of India, 2019–2021

Spatial prevalence of diarrhoea among children aged 0–5 years across 707 districts of India has been illustrated below in Fig. 1. In India, around 33% of districts (334 out of 707) have reported the prevalence of the same by more than the national average (7.3%). Around 20% (138 districts) districts have shown a prevalence between 5.3 to 7.3 percent and the remaining 47% of districts reported less than 5.3 percent of diarrhoea prevalence. Supaul and Madhubani districts from Bihar have shown the highest prevalence of diarrhoea and Kabeerdham district from Chhattisgarh and Mahe district from Puducherry have reported the lowest diarrhoea prevalence. The high prevalence districts are from the northern, western and eastern part of India mostly from Maharashtra, Bihar, Rajasthan, Gujarat, West Bengal, Odisha, Jharkhand, and Jammu and Kashmir.

**Fig. 2:**
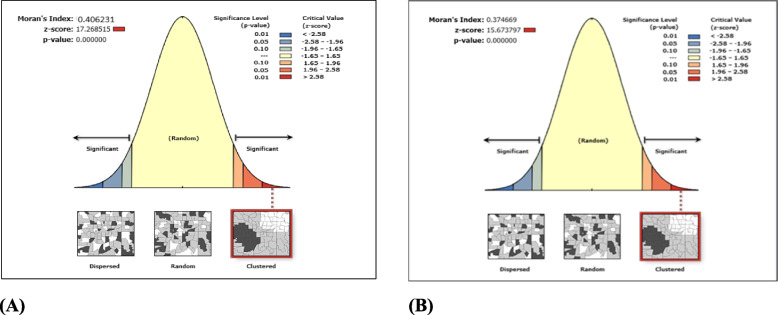
Graphical presentation of the result of spatial autocorrelation of diarrhoea prevalence among children in districts of India. **A** Graphical presentation of the result of spatial autocorrelation of diarrhoea prevalence among children in districts of India, 2019–2021. **B** Graphical presentation of the result of spatial autocorrelation of diarrhoea prevalence among children in districts of India, 2015–16

**Fig. 3:**
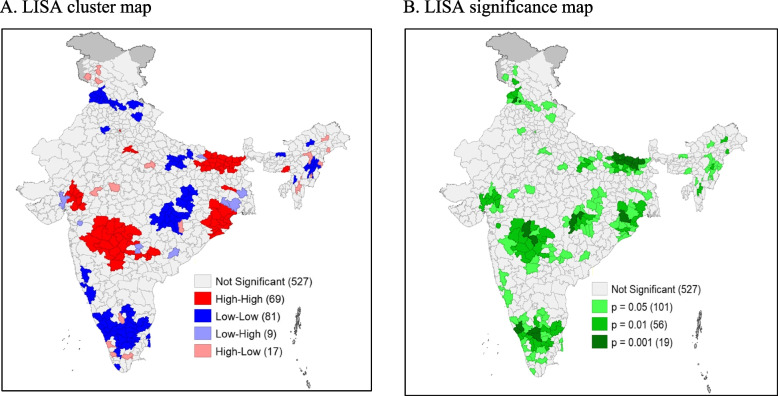
LISA cluster map and significance map for diarrhoea prevalence among children in districts of India, 2019–21 (NFHS-5)

As per NFHS -5 (2019–21), Moran’s Index is 0.41 (*p* value < 0.001) which indicates the existence of medium to high level of spatial autocorrelation in the prevalence of childhood diarrhoea across the districts in India (Fig. 2 (A)). Based on this result, the probable hot-spot districts of childhood diarrhoea prevalence in India have been identified using the LISA map. (Depicted in Figure-3). Figure 3 (A) shows the clustering of the prevalence of diarrhoea, and Fig. 3 (B) highlights the significance levels of clustering. The area of hot-spot^1^ (high to high) has been depicted in red colour. On the other hand, cold spot^2^ districts (low to low) are shown by the blue colour. A total of 69 districts can be considered under the hot-spot area and the majority of them are from the eastern and western parts of India (i.e. Maharashtra, Bihar, Odisha and Gujarat). On the other hand, a total of 81 districts falls under cold-spot areas, which are from the south, north, central, and some part of northeast India. The Significance map identified 183 districts in clusters with ≥ 5% significance level.

A comparison between the spatial prevalence to find out the change in clusters from NFHS 4 to NFHS 5.

**Fig. 4:**
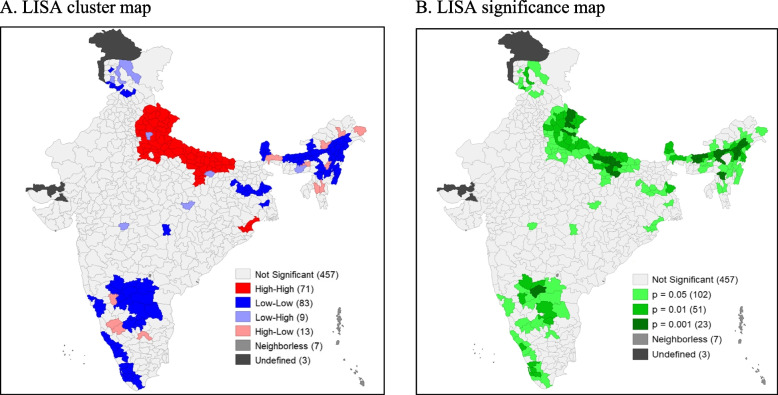
LISA cluster map and significance map for diarrhoea prevalence among children in districts of India, 2015–16. (NHFS-4)

Out of 640 districts 71 districts are detected as hot-spot areas (Fig. 4 (A)) with a Moran’s Index value of 0.38 (Fig. 2 (B)), which are mostly from Uttar Pradesh and Odisha. On the other hand, 83 districts are showing cold-spot areas from the south, north, east, and north-east parts of India. The level of significance map represents around 183 districts have reported in clusters with ≥ 5% significance level (Fig. 4 (B)).

A comparison of cluster analysis has also been done between NFHS-4 & NFHS-5. In cluster analysis across the districts, substantial changes are found. The autocorrelation has increased from NFHS-4 to NFHS-5. Moran’s Index value has increased from 0.38 to 0.41 at the same time. The new additional hot-spot districts have been detected from the eastern, western, and central parts of India (mainly from the states Maharashtra, Tamil Nādu, Bihar, Odisha and Gujarat) in 2021.

#### Background characteristics of the sample

Considering the location, around 26 percent children are from the central region followed by eastern and northern regions respectively (Table 2). About 90 percent of households have improved drinking water facilities and around 74 percent of households have improved sanitation facilities. About 80 percent of the children surveyed were from rural residences. Around 73 percent of households belong to the Hindu community and more than 42 percent are from Scheduled Tribe. About 30 percent of the household were from a category of a rich household. In the case of the mother's education, around 22 percent of women have no education, 52 percent of women have a secondary level of education, and around 14 percent have higher education (Table 2).

**Table 2:** Distribution of the sample in the study variables

**Variables Characteristics**	**Sample**	**Percent**	**Description of the Variable**
**Environmental variables**			
**Region**			
*North*	41,662	18.62	Region recorded in NFHS as North were (Chandigarh, Delhi, Haryana, HP, J & K, Panjab, Rajasthan, Uttarakhand and Ladakh), as East (Bihar, Jharkhand, Odisha, West Bengal), as North-East (Arunachal pradesh, Assam, Manipur, Meghalaya, Mizoram, Nagaland, Sikkim, Tripura), as Central (Chhattisgarh, Madhya Pradesh, Uttar Pradesh), as West (D & N Haveli, D & Diu, Goa, Gujarat, Magarastra) and as South (A & N Island, Andhra Pradesh, Karnataka, Kerala, Lakshadweep, Puducherry, Tamil Nadu, Telangana)
*East*	43,178	19.29	
*North East*	33,110	14.8	
*Central*	57,382	25.64	
*West*	19,922	8.9	
*South*	28,531	12.75	
**Type of house**			
*Kaccha*	796	0.37	Kaccha House: Houses made from mud, thatch, or other low-quality materials. Semi pucca House: houses that use partly low-quality and partly high-quality materials. pucca House: houses made with high quality materials throughout, including the floor, roof, and exterior walls, are called pucca houses
*Semi pucca*	55,427	26	
*Pucca*	1,56,964	73.63	
**Source of drinking Water**			
*Improved*	1,90,869	89.53	Improved source of drinking water: Include piped water, public taps, standpipes, tube wells, boreholes, protected dug wells and springs, rainwater, and community reverse osmosis (RO) plants. Unimproved source of drinking water: Unprotected dug well, Unprotected spring, Tanker truck/cart with small tank, Surface water, Bottled water (As per NFHS-5)
*unimproved*	22,318	10.47	
**Sanitation Facility**			
*Improved*	1,57,689	73.97	Improved sanitation facility: Include any non-shared sanitation of the following types: flush/pour flush sanitations to piped sewer systems, septic tanks, and pit latrines; ventilated improved pit (VIP)/biogas latrines; pit latrines with slabs; and twin pit/composting sanitations. Unimproved sanitation facility: Flush/pour flush not to sewer/septic tank/pit latrine, Pit latrine without slab/open pit, and Dry sanitation
*unimproved*	55,497	26.03	
**Month of Interview**			
*Rainy Session*	37,137	16.59	Rainy Session: It include May, June, July and August month. Others: January, February, March, April, September, October, November and December
*Others*	1,86,648	83.41	
**Socio-economic variables**			
**Place of Residence**			
*Urban*	45,807	20.47	Place of residence was used as original
*Rural*	1,77,978	79.53	
**Caste**			
*SC/ST*	90,814	42.86	The Indian constitution (1949) created broad categories of under privileged groups in the republic of India that were to be the object of special administrative and welfare efforts. These categories were named, though not clearly defined. SC/ST: Two groups of historically disadvantaged people recognised in constitution of India and are known as depressed classes. OBC: Other socially and educationally disadvantaged classes not included in SC/ST
*OBC*	85,656	40.42	
*Others*	35,422	16.72	
**Religion**			
*Hindu*	1,64,052	73.31	Religion of mother was used as dummy variable in three groups from original variable. Others: it includes Christians, Sikhs and Buddhists/Neo-Buddhists, Jain and others religion
*Muslim*	32,341	14.45	
*Others*	27,392	12.24	
**Wealth Index**			
*Poor*	1,12,174	50.13	In the case of wealth status, wealth quintiles I and II have been merged as 'poor' quintiles III as 'middle' and quintiles IV and V as 'rich' categories
*Middle*	43,511	19.44	
*Rich*	68,100	30.43	
**Mother’s Education**			
*No education*	48,414	21.63	Education was used as original
*Primary*	28,663	12.81	
*Secondary*	1,15,638	51.67	
*Higher*	31,070	13.88	
**Mother's Age**			
*15–24 years*	68,875	30.78	Age of the respondents at the time of survey in completed years
*25–34 years*	1,32,961	59.41	
*35–49 years*	21,949	9.81	
**Media to exposure**			
*Never*	65,445	29.24	Media Exposure was considered as ‘Everyday if respondent used any media (i.e. TV, Radio & Newspaper) for every day, considered as 'Sometimes' if media used once in a week and month and as 'Never' if no access to mass media
*Sometimes*	1,43,754	64.24	
*Everyday*	14,586	6.52	
**Child Variables**			
**Sex of Child**			
Boy	1,15,632	51.67	Sex of the child was used as original
*Girl*	1,08,153	48.33	
**Age of Child(months)**			
*0–11*	44,332	20.0	Age of the child at the time of survey in completed years
*Dec-35*	86,940	39.3	
*36–59*	89,991	40.7	
**Birth order of Child**			
*1*	85,680	38.29	Birth order categories as per children birth per women
*2*	74,051	33.09	
*3 and above*	64,054	28.62	
**WAZ-Score**			
*Normal*	1,45,343	69.11	Children whose weight-for-age Z-score is below minus two standard deviations (-2 SD) from the median of the reference population are classified as underweight. Children whose weight-for-age Z-score is below minus three standard deviations (-3 SD) from the median are considered severely underweight
*Underweight*	43,257	20.57	
Severely Underweight	21,713	10.32	

In the case of exposure to mass media, more than 64 percent of women have some exposure to mass media. The percentages of male children were around 52 percent. 38 percent of the children belong from the first order of birth. Around 22 percent of children are underweight and more than 10 percent are severely underweight (Table 2).

### The bivariate analysis of environmental, socio-economic, and child characteristics with childhood diarrhoea prevalence

**Fig. 5:**
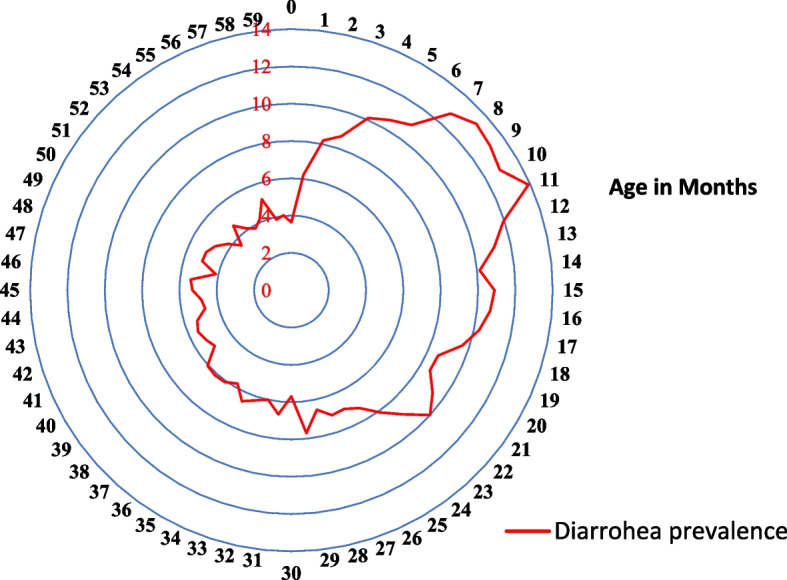
Percentage of children under age 5 who had symptoms of Diarrhoea in the two weeks preceding the survey by age

The bivariate analysis with the chi-square test for environmental, socio-economic, and child characteristics with childhood diarrhoea prevalence is presented in Table 3. The result shows that except for source of drinking water and caste group, all variables are found to be significantly (*p* < 0.001) associated with childhood diarrhoea prevalence. The analysis of data shows that out of 2,23,785 samples of children, 16,354 (7.3%) children are reported to have diarrhoea disease. The region-specific study result depicts that diarrhoea cases are higher in the western (8.3%) region as compared to other geographical regions of India. The southern region (5.4%) has shown comparatively low prevalence. Childhood diarrhoea prevalence is found to be higher among children living in kaccha households (9.7%) and households using unimproved sanitation facilities (8.6%). The prevalence is also higher in the rainy season compared to other seasons. The prevalence of diarrhoea is also higher in rural (7.7%) residents as compared to urban counterparts. Increase in the mother’s age and education decreases the prevalence. With respect to the household wealth index, children from poor households have shown higher cases of diarrhoea disease (8.6%) against middle (7.2%) and rich (5.7%) households. Further, children of less than 11 months tend to suffer from diarrhoea more compared to other age groups of children (Fig. 5). Prevalence of the disease is more among male children as compared to female children. Three and more birth order (8%), severely underweight, and underweight children have shown more vulnerability against their counterparts.

**Table 3:** Overall preview prevalence of diarrhoea and relationship of independent categorical variables of children aged 0–5 in India, 2019–21

Predictors	Prevalence of Diarrhoea (Yes)		Chi-square *p* value
**Prevalence of Diarrhoea**^a^	***N***	***%***	
No	2,07,431	92.69	NA
Yes	16,354	7.31	
**Environmental variables**			
**Region**			0.000
North	1,821	6.04	
East	6,160	10.57	
North East	487	5.96	
Central	3,452	5.61	
West	2,425	8.62	
South	2,009	5.35	
**Type of house**			0.000
*Kaccha*	80	9.71	
*Semi pucca*	4380	7.82	
*Pucca*	11,015	7.05	
**Source of drinking Water**			
*Improved*	14,169	7.29	0.242
*unimproved*	1,305	6.97	
**Sanitation facility**			0.000
*Improved*	10,568	6.77	
*unimproved*	4,907	8.59	
**Month of Interview**			0.000
*Rainy Session*	4,565	10.26	
*Others*	11,788	6.57	
**Socio-economic variables**			
**Place of Residence**			0.000
*Urban*	3,714	6.17	
*Rural*	12,640	7.73	
**Caste**			
*SC/ST*	5,633	7.6	0.062
*OBC*	6,942	7.11	
*Others*	2,981	7.42	
**Religion**			0.000
*Hindu*	13,022	7.33	
*Muslim*	2,729	7.5	
*Others*	603	6.16	
**Wealth Index**			0.000
*Poor*	8,735	8.57	
*Middle*	3,173	7.24	
*Rich*	4,446	5.74	
**Mother’s Education**			0.000
*No education*	3,664	7.81	
*Primary*	2,168	7.94	
*Secondary*	8,611	7.56	
*Higher*	1,910	5.36	
**Mother's Age**			0.000
*15–24 years*	6,650	9.05	
*25–34 years*	8,584	6.51	
*35–49 years*	1,120	6.07	
**Media to exposer**			0.000
*Never*	5,148	8.09	
*Sometimes*	10,275	7.06	
*Everyday*	931	6.39	
**Child Variables**			
**Sex of Child**			0.000
Boy	8,773	7.56	
*Girl*	7,580	7.03	
**Age of Child (Months)**			0.000
*0–11*	4,600	10.37	
*12–35*	7,302	8.38	
*36–59*	4,330	4.83	
**Birth order of Child**			0.001
*1*	6,119	6.98	
*2*	5,414	7.14	
*3 and above*	4,821	7.99	
**WAZ Score**			0.000
*Normal*	10,234	7.16	
*Underweight*	3,612	8	
Severely Underweight	1,855	8.31	

Source: National Family Health Survey (NFHS)-5, 2019–21

*N* number, *%* Percentage; Prevalence of Diarrhoea: Row percentage. Chi-square test: *p* < .05 Significance; *p* > 0.05 Not significant

^a^Diarrhoea Prevalence: 0 = not suffered from diarrhoea, 1 = suffered from diarrhoea

Among environmental factors (Table 4), it has been found that children from the eastern region [OR = 1.44, 95% CI (1.24–1.67)] of India are more likely to suffer from diarrhoea than those from the northern region, whereas the southern region [OR = 0.86, 95% CI (0.74–1.0)] is less likely to have diarrhoea as compared to the northern region. The odds of children who are living in the pucca house [OR = 0.89, 95% CI (0.68–1.16)] being less likely to suffer from diarrheal disease as compared to those living in the kaccha house.

**Table 4:** Estimated odds ratio obtained from logistic regression models of diarrhoea among 0–5 aged children in India, 2019–2021

**Predictors**	**AOR [95% CI]**	***p*-value**
**Environmental variables**		
**Region**		
*North*	*Reference*	NA
*East*	1.44 [1.24–1.67]	0.000
*North East*	0.79 [0.67–0.93]	0.005
*Central*	0.88 [0.77–1.01]	0.067
*West*	1.23 [1.04–1.46]	0.022
*South*	0.87 [0.75–1.01]	0.054
**Type of house**		
*Kaccha*	*Reference*	NA
*Semi pucca*	0.89 [0.68–1.15]	0.346
*Pucca*	0.89 [0.68–1.15]	0.049
**Source of drinking Water**		
*Improve*	*Reference*	NA
*unimproved*	0.96 [0.9–1.02]	0.139
**Sanitation facility**		
*Improved*	*Reference*	NA
*unimproved*	1.02 [0.98–1.07]	0.468
**Month of Interview**		
*Rainy Session*	*Reference*	NA
*Others*	0.73 [0.66–0.8]	0.000
**Socio-economic variables**		
**Place of Residence**		
*Urban*	*Reference*	NA
*Rural*	1.01 [0.95–1.06]	0.918
**Caste**		
*SC/ST*	*Reference*	NA
*OBC*	0.93 [0.89–0.98]	0.001
*Others*	0.91 [0.86–0.97]	0.001
**Religion**		
*Hindu*	*Reference*	NA
*Muslim*	1.04 [0.98–1.11]	0.259
*Others*	0.96 [0.87–1.06]	0.371
**Wealth Index**		
*Poor*	*Reference*	NA
*Middle*	0.98 [0.93–1.04]	0.372
*Rich*	0.91 [0.86–0.97]	0.003
**Mother’s Education**		
*No education*	*Reference*	NA
*Primary*	1.18 [1.11–1.26]	0.000
*Secondary*	1.15 [1.09–1.22]	0.000
*Higher*	1.0 [0.93–1.09]	0.980
**Mother's Age**		
*15–24 years*	*Reference*	NA
*25–34 years*	0.89 [0.85–0.93]	0.000
*35–49 years*	0.9 [0.83–0.98]	0.007
**Media to exposure**		
*Never*	*Reference*	NA
*Sometimes*	1.1 [1.05–1.15]	0.000
*Everyday*	1.07 [0.98–1.16]	0.188
**Child Variables**		
Sex of Child		
Boy	*Reference*	NA
*Girl*	0.93 [0.9–0.97]	0.000
**Age of Child(months)**		
*0–11*	*Reference*	NA
*12–35*	0.82 [0.78–0.85]	0.000
*36–59*	0.48 [0.46–0.5]	0.000
**Birth order of Child**		
*1*	*Reference*	NA
*2*	1.02 [0.98–1.07]	0.398
*3 and above*	1.05 [0.99–1.11]	0.140
**WAZ score (Underweight)**		
*Normal*	*Reference*	NA
*Underweight*	1.08 [1.03–1.13]	0.001
*Severely Underweight*	1.05 [0.99–1.12]	0.123
**Overall Model Evaluation**		
*Observation (N)*	189,915	
*Log likelihood (model)*	-45,467.78	
*Degrees of freedom*	33	
*Wald Chi2*	1415.31	
*P-value (Chi2)*	0.000	
*ICC [95% CI]*	0.077 [0.068, 0.088]	
*SD [SE]*	0.52 [0.02]	

*AOR* Adjusted Odd Ratio, *CI* Confidence interval, *p* < *.05* Significance

*ICC* intra-cluster correlation coefficient, *SD* Standard deviation, *SE* Standard error

Diarrhoea: 0 = not suffered from diarrhoea, 1 = suffered from diarrhoea

In the case of social groups, the odds of children suffering from diarrhoea are less likely among OBC [OR = 0.93, 95% CI (0.89–0.98)]. and others category [OR = 0.91, 95% CI (0.86–0.97)] as compared to the SC/ST category.

Children of rich households [OR = 0.91, 95% CI (0.86–0.97)] are markedly less likely to experience diarrhoea as compared to children of poor households.

Moreover, children of mothers with primary [OR = 1.18, 95% CI (1.11–1.26)] and secondary [OR = 1.15, 95% CI (1.09–1.22)] education are more likely to suffer from diarrhoea as compared to children of non-educated mothers. The likelihood of experiencing diarrhoea has decreased as the mother's age has increased (Table 4).

The odds of children suffering from diarrhoea among female children [OR = 0.93, 95% CI (0.9–0.97)] are less likely compared to male children. With the increase in child’s age, the risk of such morbidity is reduced. In terms of child nutrition status, underweight children [OR = 1.08, 95% CI (1.03–1.13)] are more likely than normal-weight children to suffer from diarrheal diseases.

## Discussion

Diarrhoea is one of the leading public health problems specially in low- and middle-income countries like India and one of the killer diseases specially among children under five years. The integrated Global Action Plan for the Prevention and Control of Pneumonia and Diarrhoea (GAPPD) introduces many cohesive approaches to prevent child morbidity and mortality due to pneumonia and diarrhoea, [19]. However, due to the low coverage of programmes and poor implementation, it is not achieved in most countries, including India [25–28]. The present study examines the prevalence and spatial clustering along with the factors associated with childhood diarrhoea across the districts of India in the year 2019–21. This is the first comprehensive study to discuss the spatial autocorrelation along with factors associated with childhood diarrhoea in India among under five children by using National Family Health Survey-5 (2019–21) data. It shows overall, 7.3 percent of children suffer from diarrhoeal diseases in the country. The prevalence has been reduced by around 2 percent from 2016 to 2019 in the country [15, 16].

Several programmes and strategies have been launched by the Indian government to reduce child mortality in order to achieve Millennium Development Goal 4. There have been millions of individuals impacted by programs such as the Child Survival and Safe Motherhood Program (1992), the Target-Free Approach (1996), the Reproductive and Child Health Program (1997), the National Rural/Urban Health Mission (2005–2012), and the National Health Mission (since 2013) [15]. These programmes covered vaccine campaigns, diarrhoea prevention, acute respiratory infections (ARI) prevention etc. [15]. The under-five (U-5) mortality has also decreased from 55/1000 live births to 42/1000 live births between 2011 to 2021[13–15, 29]. Still, the prevalence of diarrhoea especially among under five children requires further attention especially for high prevalence regions. As our findings demonstrate the prevalence of diarrhoea is highest in Bihar (13.7%) and lowest in Lakshadweep (2.3%). The findings by other researchers also show various health care program and schemes did not significantly improve health-care providers’ knowledge or performance with regard to childhood diarrhoea or pneumonia in Bihar [30]. A Study by Kumar et. al, (2015) also shows that the overall use of zinc was low in Bihar. Zinc supplementation along with oral rehydration solutions not only help to reduce the duration of diarrheal diseases but also the severity of diarrheal episodes [31].

The spatial analysis shows around 33% of districts which are having more than the national level diarrhoeal diseases are mostly from eastern, western, and northern parts of the country, in the period 2019–2021. If we look at the analysis based on NFHS-4 data, a mild spatial autocorrelation is still existing in India in the period of 2019–21 [15]. A significant (*p* < 0.01) Moran’s Index (0.41) value with 69 hot-spot districts demonstrates a medium to a high-level of autocorrelation exists in the prevalence of childhood diarrhoea. The hotspot of diarrhoea changed from Uttar Pradesh to Maharashtra from NFHS-4 (2015–16) to NFHS-5 (2019–21). The possible reason is that the prevalence in Maharashtra has been increased from NFHS-4 to NFHS-5 while, the prevalence has decreased in Uttar Pradesh in the same period. As a high level of prevalence across the neighbourhood districts was detected in Maharashtra, so by definition we can call it as a “hot-spot cluster”. Moreover, the prevalence of childhood diarrhoea is higher than the national average in the state of Maharashtra. Prevalence has increased from 8.5 percent to 8.9 percent between 2016 and 2021 and around 50 percent of districts showed increasing prevalence for childhood diarrhoea [32]. The prevalence of the diseases in Uttar Pradesh has decreased from 15 percent to 5.57 percent in the same period. Due to the decrease in prevalence from NFHS-4 to NFHS-5, the cluster has disappeared from Uttar Pradesh.

The present study also identifies the factors associated with childhood diarrhoeal diseases. Its occurrence is correlated with the characteristics and infrastructural facilities of the house. Children living in kuccha houses are more likely to suffer from diarrhoeal diseases. Similar findings in various studies [15, 31, 33, 34].

The study has found an insignificant relationship between an improved source of drinking water and childhood diarrhoea prevalence, though previous researchers have found a significant link between the two [15, 35–37].

We have found children belonging to rich households are markedly in lesser risk to experience diarrhoea, which resembles similar findings in contemporary literature [15, 16]. Surprisingly, we have found mother’s higher education increases the risk of occurrence which has been also found by other researchers [15, 16]. Our finding on the influence of mother’s age on the occurrence of childhood diarrhoea is also similar to what has been portrayed in contemporary research [15, 17, 38–40]. One probable explanation is that women who are qualified and knowledgeable about this disease tend to remember and report more accurately [15, 16].

We have also found children within the age group 12–34 months and > 35 months have a lesser occurrence of diarrhoea as compared to 0–12 monthschildren. This finding is relatable to the current studies [35, 41–44]. Our findings also show underweight children are at higher risk of suffering from diarrhoeal diseases as compared to normal weight children. A Study published in Lancet reported that diarrhoea episodes are positively associated with childhood growth and also found that each day of diarrhoea prevalence increases with increasing WAZ score [45].

### Policy implication

The geo-spatial analysis shows that a medium to a high level of autocorrelation has been detected from 2016 to 2021 for childhood diarrhoea across the districts in India. As we know, spatial autocorrelation in health events may be the sign of underlying causal factors leading to the localization of disease clusters; it is a serious public health concern in the country. Overall hot-spots are detected from Uttar Pradesh, Maharashtra, Tamil Nadu, Bihar, Odisha and, Gujarat. The highlighted hot-spot districts can help to make a district level policy to prevent the prevalence. The Government of India has identified around 125 districts (*In Phase I:*115 & Phase II: 9) as aspirational districts (https://my.msme.gov.in/MyMsme/List_of_AspirationalDistricts.aspx). Most of the aspirational districts are Jharkhand, Bihar, Uttar Pradesh, Odisha, and Maharashtra. The cluster maps for the last two rounds of NFHS are also showing that most of the hot-spot districts belong to those states only. A further study is needed for comparison with high prevalence as well as hot-spot districts with aspirational districts that can give more pictorial findings for childhood diarrhoea.

Most of our findings related to the determinants of diarrhoea among children are similar to the findings in the contemporary literature. Among infrastructural indicators, as a determinant of childhood diarrhoea the most important is the type of household (“kuccha” or “concrete”) and the drinking water facility. We know, there exists a gap between supply and demand for concrete housing mainly for the middle-class population. The Ministry of Housing & Urban Poverty Alleviation also estimated such demand resulted from housing shortages for the entire country from 2012–2016. The Government has also taken various steps to help the ‘poor’ to construct houses (like Indira Awas Yojona, the Slum Rehabilitation Authority project). But sometimes the targeted beneficiaries do not have the ability to apply for these benefits without the assistance of middlemen or the direct intervention of government officials [23, 46]. Local bodies need to get involved in community development through innovative schemes, which may be supported by a private–public partnership for healthy living [23]. However, the Government of India implemented Swacha Bharat Mission Schemes to improve the situation but many households still do not proper sanitation facility. Therefore, the government should implement further policies for the better availability of sanitation and drinking water facility. Our findings on the impact of mother’s age on children’s diarrhoea show the need for higher education among women.

#### Limitations of the study

The present study has some limitations. Firstly, the responses pertaining to diarrhoea diseases (last two weeks preceding the survey), were self-reported and completely based on the mother’s knowledge, therefore prone to recall bias. Secondly, it is not medically verified during the survey, hence, may not be true for generalizing overall study findings. Lastly, the study didn’t include variables like dietary diversity, immunization, and breastfeeding practices that may play important roles, and hence, a further study may utilize information on those aspects while explaining the prevalence of diarrhoea among under five children in India.

## Conclusion

The present study based on NFHS-5 (conducted in 2019–21) not only identifies the high prevalence and hot-spot districts of childhood diarrhoea in India but also the factors associated with the disease. The geo-spatial analysis shows that medium to high levels of autocorrelation was detected from 2016 to 2021 for childhood diarrhoea across the districts in India. The study has found that the mother’s age, and improvement in sanitation facilities can reduce the risk of the disease among children. Moreover, children, those who are living in kuccha houses, suffering from malnutrition are atrisk of childhood diarrhoea. Based on findings, the study recommends that the public health programmes can implement a district level policy by targeting hot spot districts where the higher prevalence of diarrhoea is persisting and can implement needful preventive measures to control the incident and burden of childhood diarrhoea in India.

## Data Availability

The data are collected from DHS (Demographic Health Survey), which is available publicly. The data can be downloaded via online through DHS website. https://dhsprogram.com/data/

## Abbreviations

SDGs: Sustainable development Goals
WHO: World Health Organisation
UNICEF: United Nations International Children’s Emergency Fund
NFHS: National Family health Survey
GAPPD: Global Action Plan for Pneumonia and Diarrhoea
MoHFW: Ministry of Health & Family Welfare
IIPS: Institute for Population Sciences
CAB: Clinical and Biochemical
ESDA: Exploratory Spatial Data Analysis
GIS: Geographical Information System
LISA: Local Indicators of Spatial Autocorrelation
CI: Confidence Interval

## Footnotes

1 Districts with a higher prevalence of diarrhoea are surrounded by neighbouring districts with greater levels of diarrhoea.

2 Districts with lower levels of diarrhoea prevalence and have similar neighbourhoods.

## References

[1] Troeger C, Blacker B, Khalil IA, Rao PC, Cao J, Zimsen SR, Albertson SB, Deshpande A, Farag T, Abebe Z (2018). GBD 2016 Lower Respiratory Infections Collaborators Estimates of the global, regional, and national morbidity, mortality, and aetiologies of lower respiratory infections in 195 countries, 1990–2016: a systematic analysis for the Global Burden of Disease Study 2016. Lancet Infect Dis.

[2] World Health Organization. Causes of child death. https://www.who.int/data/gho/data/themes/topics/indicator-groups/indicator-group-details/GHO/causes-of-child-death. Accessed on 15 May 2022.

[3] World Health Organization. Fact sheet: Diarrhoeal disease.https://www.who.int/news-room/fact-sheets/detail/diarrhoeal-disease Accessed on 15 May 2022.

[4] United Nations Children’s Fund. Levels and trends of Child mortality: by United Nations Inter-agency Group for Child Mortality Estimation. UNICEF, 2019. https://www.unicef.org/media/60561/file/UN-IGME-child-mortality-report-2019.pdf. Accessed on 15 May 2022.

[5] Walker CL, Rudan I, Liu L, Nair H, Theodoratou E, Bhutta ZA, O'Brien KL, Campbell H, Black RE (2013). Global burden of childhood pneumonia and diarrhoea. The Lancet.

[6] Anand K, Sundaram KR, Lobo J, Kapoor SK (1994). Are diarrheal incidence and malnutrition related in under five children? A longitudinal study in an area of poor sanitary conditions. Indian Pediatr.

[7] Black RE, Cousens S, Johnson HL, Lawn JE, Rudan I, Bassani DG, Jha P, Campbell H, Walker CF, Cibulskis R, Eisele T (2010). Global, regional, and national causes of child mortality in 2008: a systematic analysis. The lancet.

[8] Ghosh K, Gupta SS, Chakraborty AS. Childhood Morbidity and its Association with Socio-economic and Health Care Condition among Under 5 Years Children in West Bengal: An Evidence from NFHS-5, 2019–20. Int J Med Public Health. 2021;11(3):160–3.

[9] Shah D, Choudhury P, Gupta P, Mathew JL, Gera T, Gogia S, Mohan P, Panda R, Menon S (2012). Promoting appropriate management of diarrhea: a systematic review of literature for advocacy and action: UNICEF-PHFI series on newborn and child health, India. Indian Pediatr.

[10] MoHSW. (2013a). National guideline of infant and young child feeding. Ministry of Health and Social Welfare, 1–120. https://wcd.nic.in/sites/default/files/nationalguidelines.pdf Accessed on 15 May 2022.

[11] International Institute for Population Sciences. National Family Health Survey (NFHS-4), 2015–16, India Fact Sheet. Mumbai: IIPS. 2017. http://rchiips.org/nfhs/factsheet_NFHS-4.shtml Accessed on 29 May 2022.

[12] International Institute for Population Sciences. National Family Health Survey (NFHS-3), 2005–06, India Fact Sheet. Mumbai: IIPS, 2006. http://rchiips.org/nfhs/factsheet_NFHS-4.shtml Accessed on 29 May 2022.

[13] International Institute for Population Sciences. National Family Health Survey (NFHS-3), 2019–21, India Fact Sheet. Mumbai: IIPS, 2019–21. http://rchiips.org/nfhs/factsheet_NFHS-4.shtml. Accessed on 29 May 2022.

[14] Lakshminarayanan S, Jayalakshmy R (2015). Diarrheal diseases among children in India: current scenario and future perspectives. J Nat Sci Biol Med.

[15] Ghosh K, Chakraborty AS, Mog M (2021). Prevalence of diarrhoea among under five children in India and its contextual determinants: a geo-spatial analysis. Clin Epidemiol Global Health.

[16] Mallick R, Mandal S, Chouhan P (2020). Impact of sanitation and clean drinking water on the prevalence of diarrhea among the under-five children in India. Child Youth Serv Rev.

[17] Edwin P, Azage M (2019). Geographical variations and factors associated with childhood diarrhea in Tanzania: a national population based survey 2015–16. Ethiop J Health Sci.

[18] Sustainable Development Goal (SDGs): https://www.undp.org/content/undp/en/home/sustainable-development-goals/goal-3-good-health-and-well-being.html Accessed on 22 May 2022.

[19] World Health Organization. The integrated Global Action Plan for Prevention and Control of Pneumonia and Diarrhoea (GAPPD): Ending two major preventable causes of child death. https://www.who.int/publications/i/item/the-integrated-global-action-plan-for-prevention-and-control-of-pneumonia-and-diarrhoea-(gappd) Accessed on 22 May 2022.

[20] Anselin L. Spatial econometrics: methods and models. Springer Science & Business Media; 1988; Rev. 47. p. 777–78.

[21] Ghosh K, Dhillon P, Agrawal G (2020). Prevalence and detecting spatial clustering of diabetes at the district level in India. J Public Health.

[22] Azage M, Kumie A, Worku A, Bagtzoglou AC (2016). Childhood diarrhea in high and low hotspot districts of Amhara Region, northwest Ethiopia: a multilevel modeling. J Health Popul Nutr.

[23] Chattopadhyay A, Barnwal A. How far environmental factors are related with acute respiratory diseases and diarrhea among young children in India. Indian J Matern Child Health. 2013;15(4):1–17.

[24] Parvin F, Ali SA, Hashmi S, Ahmad A (2021). Spatial prediction and mapping of the COVID-19 hotspot in India using geostatistical technique. Spat Inf Res.

[25] Caballero MT, Bianchi AM, Nuño A, Ferretti AJ, Polack LM, Remondino I, Rodriguez MG, Orizzonte L, Vallone F, Bergel E, Polack FP (2019). Mortality associated with acute respiratory infections among children at home. J Infect Dis.

[26] Selvaraj K, Chinnakali P, Majumdar A, Krishnan IS (2014). Acute respiratory infections among under-5 children in India: a situational analysis. J Nat Sci Biol Med.

[27] Million Death Study Collaborators (2010). Causes of neonatal and child mortality in India: a nationally representative mortality survey. Lancet.

[28] Vashishtha VM (2010). Current status of tuberculosis and acute respiratory infections in India: much more needs to be done!. Indian Pediatr.

[29] Chaikaew N, Tripathi NK, Souris M (2009). Exploring spatial patterns and hotspots of diarrhea in Chiang Mai, Thailand. Int J Health Geogr.

[30] Mohanan M, Giardili S, Das V, Rabin TL, Raj SS, Schwartz JI, Seth A, Goldhaber-Fiebert JD, Miller G, Vera-Hernandez M (2017). Evaluation of a social franchising and telemedicine programme and the care provided for childhood diarrhoea and pneumonia, Bihar, India. Bull World Health Organ.

[31] Kumar S, Roy R, Dutta S (2015). Scaling–up public sector childhood diarrhea management program: lessons from Indian states of Gujarat, Uttar Pradesh and Bihar. J Glob Health.

[32] Ghosh K, Chakraborty AS, Mog M, Zakir S (2021). Prevalence of diarrhoea and acute respiratory infection among under five children: a spatial-temporal changes of Maharashtra Districts. EC Paediatrics.

[33] Arnold BF, Colford JM (2007). Treating water with chlorine at point-of-use to improve water quality and reduce child diarrhea in developing countries: a systematic review and meta-analysis. Am J Trop Med Hyg.

[34] Kuberan A, Singh AK, Kasav JB, Prasad S, Surapaneni KM, Upadhyay V, Joshi A (2015). Water and sanitation hygiene knowledge, attitude, and practices among household members living in rural setting of India. J Nat Sci Biol Med.

[35] Nandi A, Megiddo I, Ashok A, Verma A, Laxminarayan R (2017). Reduced burden of childhood diarrheal diseases through increased access to water and sanitation in India: a modeling analysis. Soc Sci Med.

[36] Kumar A, Das KC (2014). Drinking water and sanitation facility in India and its linkages with diarrhoea among children under five: evidences from recent data. Int J Humanit Soc Sci Invent.

[37] Kumar S, Vollmer S (2013). Does access to improved sanitation reduce childhood diarrhea in rural India?. Health Econ.

[38] Ghasemi AA, Talebian A, MasoudiAlavi N, Moosavi GA (2013). Knowledge of mothers in management of diarrhea in under-five children, in Kashan, Iran. Nurs Midwifery Stud.

[39] Boadi KO, Kuitunen M (2005). Childhood diarrheal morbidity in the Accra Metropolitan Area, Ghana: socio-economic, environmental and behavioral risk determinants. J Health Popul Dev Ctries.

[40] Yassin K (2000). Morbidity and risk factors of diarrheal diseases among under-five children in rural Upper Egypt. J Trop Pediatr.

[41] Fuller JA, Westphal JA, Kenney B, Eisenberg JN (2015). The joint effects of water and sanitation on diarrhoeal disease: a multicountry analysis of the D emographic and H ealth S urveys. Tropical Med Int Health.

[42] Paul P, Mondal D (2020). Maternal experience of intimate partner violence and its association with morbidity and mortality of children: Evidence from India. PLoS One.

[43] Kamath A, Shetty K, Unnikrishnan B, Kaushik S, Rai SN (2018). Prevalence, patterns, and predictors of diarrhea: a spatial-temporal comprehensive evaluation in India. BMC Public Health.

[44] SinmegnMihrete T, AsresAlemie G, Shimeka TA (2014). Determinants of childhood diarrhea among underfive children in Benishangul Gumuz regional state, north West Ethiopia. BMC Pediatr.

[45] Troeger C, Colombara DV, Rao PC, Khalil IA, Brown A, Brewer TG, Guerrant RL, Houpt ER, Kotloff KL, Misra K, Petri WA (2018). Global disability-adjusted life-year estimates of long-term health burden and undernutrition attributable to diarrhoeal diseases in children younger than 5 years. Lancet Glob Health.

[46] Knowledge I. India’s Rural Poor: Why Housing Isn’t Enough to Create Sustainable Communities’. Wall Street Journal. 2007 Jul;7. https://knowledge.wharton.upenn.edu/article/indias-rural-poor-why-housing-isnt-enough-to-create-sustainable-communities/ . Accessed:25 June 2022

